# Supplementary figures and images for: son is necessary for proper vertebrate blood development
Source: PLoS One. 2021 Feb 25;16(2):e0247489. doi: 10.1371/journal.pone.0247489 (PMC7906411; doi:10.1371/journal.pone.0247489)

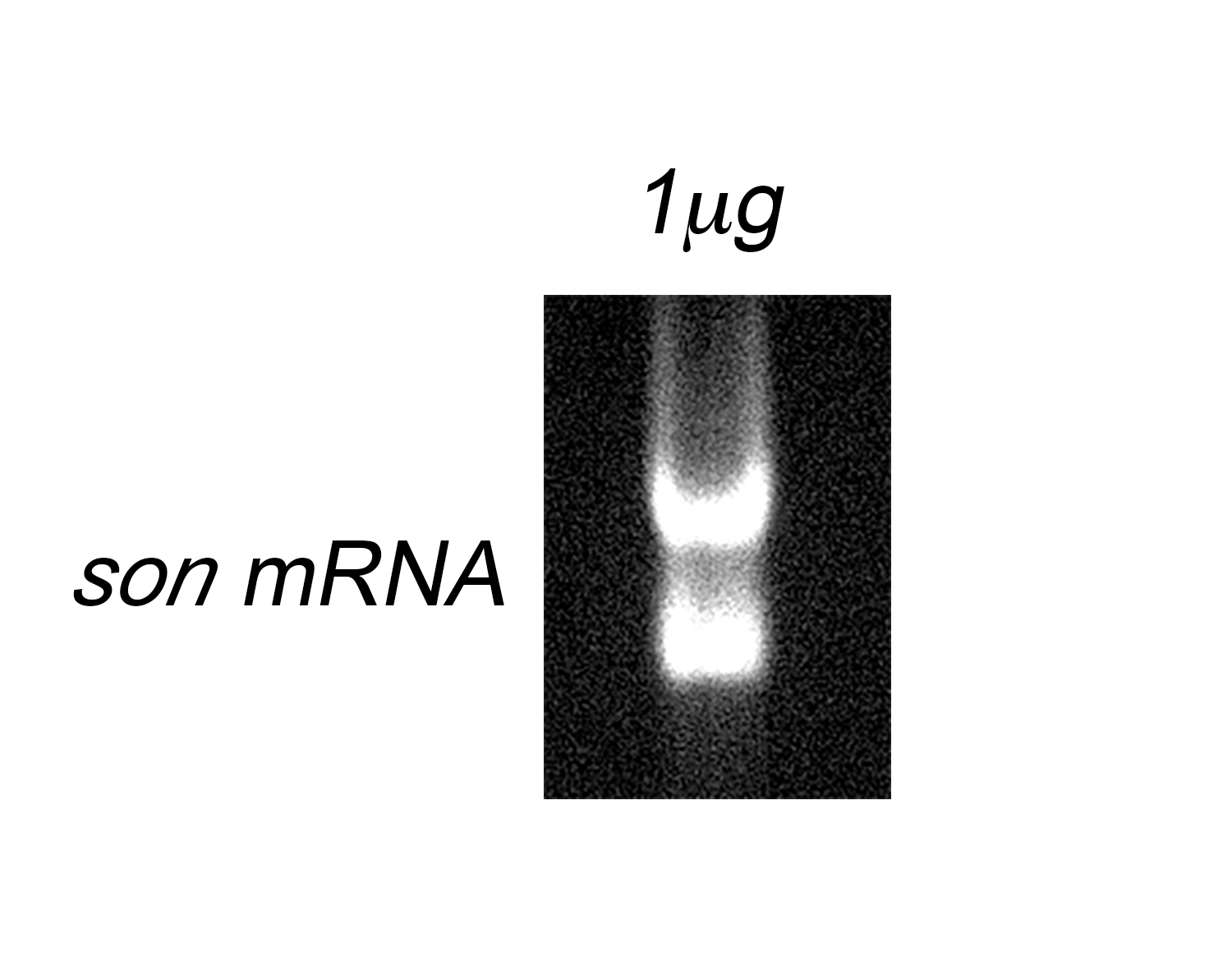

Supplement: S1 Fig — Full length son mRNA produced by the mMachine kit has two sizes: the top band is mRNA produced from circular son plasmid, and the bottom band is from linearized son plasmid. (TIF) [file pone.0247489.s001.tif]

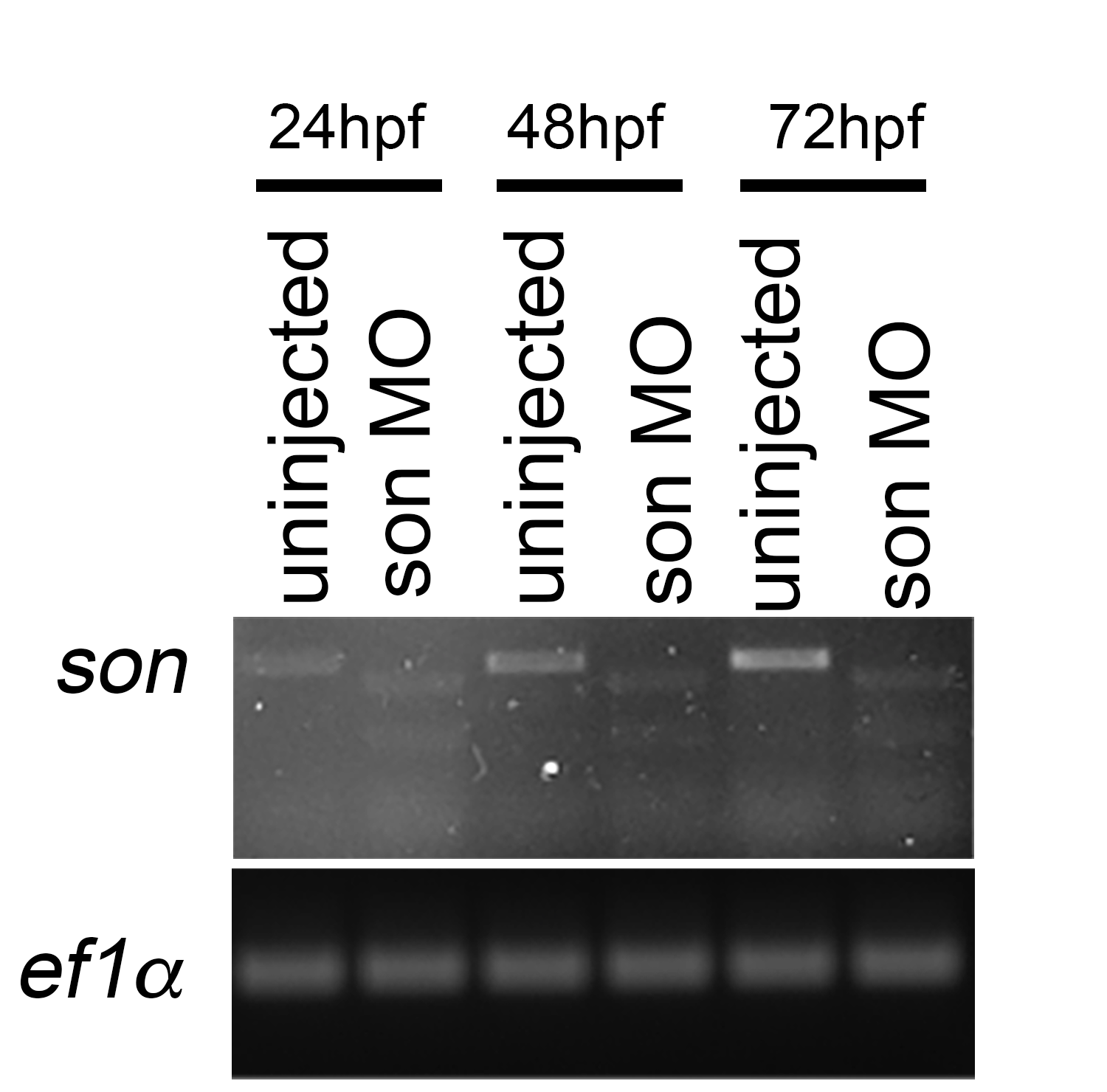

Supplement: S2 Fig — 10 random embryos either uninjected or injected with son MO at the one-cell-stage of development (son MO) collected at 24hpf (left), 48hpf (middle), and 72hpf (right) were subjected to RT-PCR for son (top) and ef1α (bottom) transcripts. (TIF) [file pone.0247489.s002.tif]

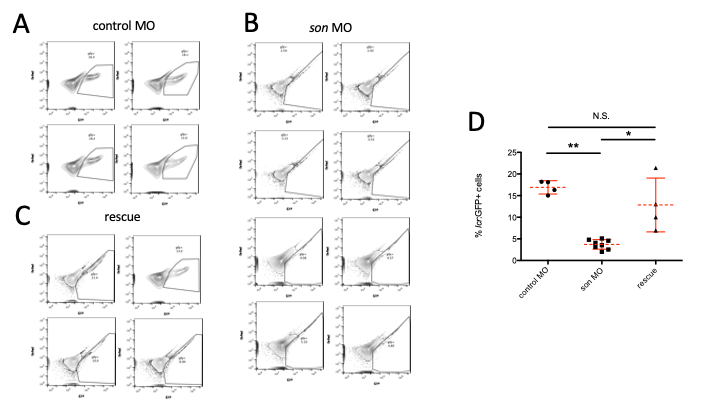

Supplement: S3 Fig — Flow cytometry of lcr:GFP+ embryos at 48hpf either injected with control MO (A), son MO (B), or MO-injected with son RNA (rescue) (C). lcr:GFP is shown along the x-axis; y-axis is the red fluorescence channel, examined at the same time to gate out auto-fluorescent and light-refractive cells in the developing embryo. Each plot represents ten embryos randomly selected from control MO, MO-injected (son MO), and MO-injected with son RNA (rescue) conditions that were enzymatically dissociated and analyzed with a flow cytometer. The numbers in the gates are the percentage of GFP+ RBCs present. (D) Data presented in graphical format. Middle lines represent mean and error bars represent SD. * represents p = 0.05, ** represents p < 0.001, N.S. represents no significance. (TIFF) [file pone.0247489.s003.tiff]

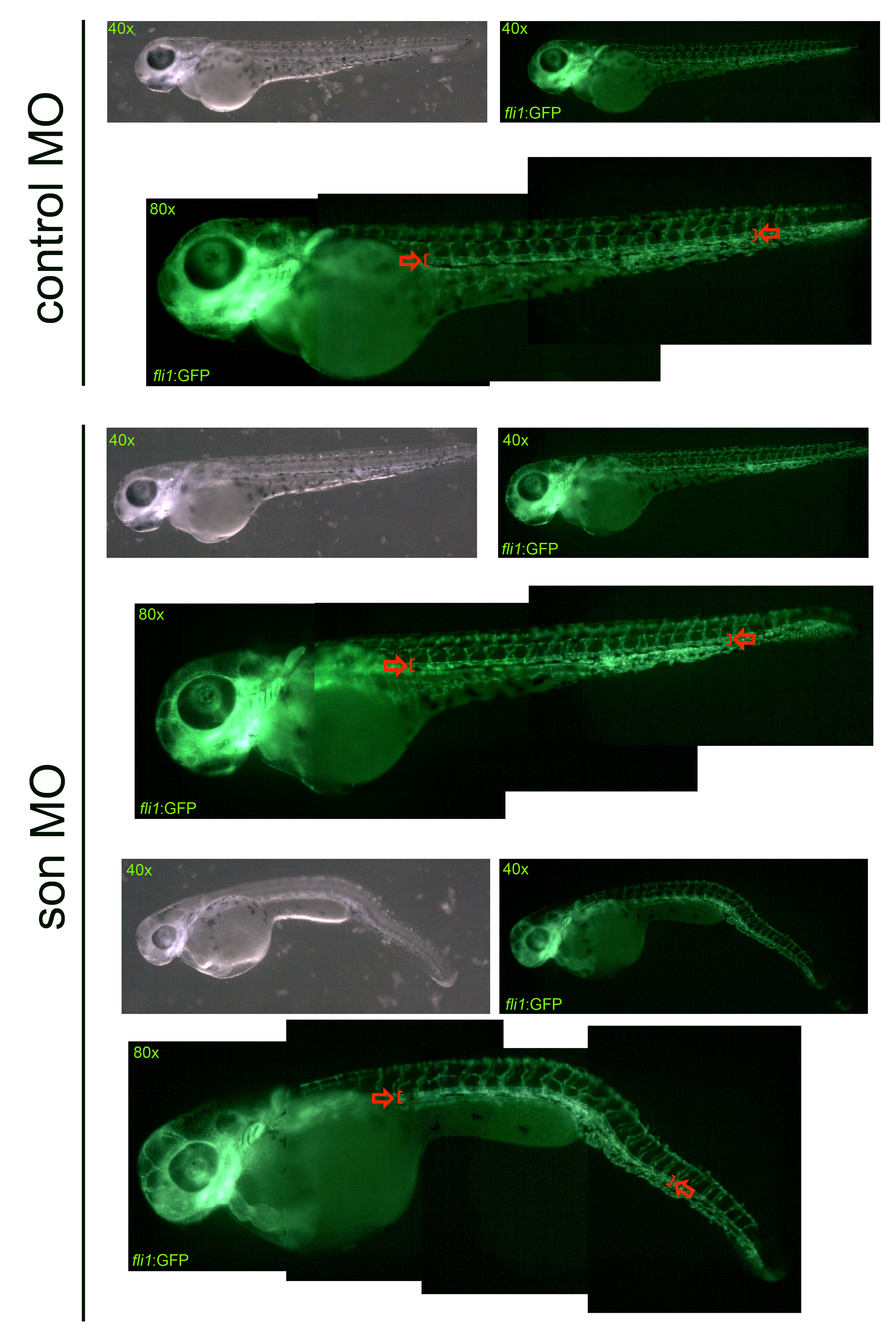

Supplement: S4 Fig — Representative images of 48hpf fli1:GFP embryos injected with control MO (top) or son MO (bottom) at the one-cell-stage of development show no significant differences in vessel formation or integrity associated with son MO injection. Brightfield images are shown in top left (40x), and fluorescent images are shown in top right (40x). Zoomed in fluorescent images are shown below (80x). Red arrows and brackets indicate the dorsal aorta, the site of HSC formation. (TIF) [file pone.0247489.s004.tif]
